# Septoria blotch epidemic process on spring wheat varieties

**DOI:** 10.18699/VJ20.609

**Published:** 2020-03

**Authors:** E.Yu. Toropova, O.A. Kazakova, V.V. Piskarev

**Affiliations:** Novosibirsk State Agrarian University, Novosibirsk, Russia All-Russian Research Institute of Phytopathology, r. p. Bolshie Vyazemy, Odintsovo district, Moscow region, Russia; Novosibirsk State Agrarian University, Novosibirsk, Russia All-Russian Research Institute of Phytopathology, r. p. Bolshie Vyazemy, Odintsovo district, Moscow region, Russia; Siberian Research Institute of Plant Production and Breeding – Branch of the Institute of Cytology and Genetics of Siberian Branch of the Russian Academy of Sciences, Novosibirsk, Russia

**Keywords:** Septoria leaf and ear blotch, spring wheat, monitoring, Рarastagonospora nodorum, Septoria tritici, P. avenae f. sp. triticae, variety, resistance, seed transmission, септориоз листьев и колоса, яровая пшеница, мониторинг, Рarastagonospora nodorum, Septoria tritici, P. avenae f. sp. triticae, сорт, устойчивость, семенная передача

## Abstract

The Septoria blotch of spring wheat leaves and ears is one of the most economically significant infections in the Siberian region. In the control systems of Septoria blotch the main ecologically safe element is resistant varieties, which are designed to slow down the pathogens reproduction rate and slow down or stop the development of the epiphytotic process. The purpose of the work was to clarify the species composition of Septoria blotch pathogens for West Siberian regions and spring wheat varieties, to study the epiphytotic process of Septoria differentially on the leaves and ears of varieties, and to evaluate the activity of seed transmission of Parastagonospora nodorum. Studies were carried out in 2016–2018 according to generally accepted methods. Septoria leaf and ear blotch of spring wheat is widespread in West Siberia and the Trans-Urals, causing a decrease in yield by up to 50 % or more with the deterioration in grain quality. The causative agents of the disease are P. nodorum, Septoria tritici, and P. avenae f. sp. triticae, and the species ratio varied across the regions and varieties, and within plant organs. In Novosibirsk Region, P. nodorum completely dominated; S. tritici was 13.8 times less common; and P. avenae f. sp. triticae was a singleton. In Tyumen Region, the dominance of P. nodorum was disrupted in some geographic locations by S. tritici and P. avenae f. sp. triticae. In Altai Krai, P. nodorum predominated at all points studied; S. tritici and P. avenae f. sp. triticae were found everywhere, but 5.6 and 8.6 times less often, respectively. The study of spring wheat varieties of different origins has not revealed any samples immune to Septoria blotch. A differentiated manifestation of resistance to Septoria leaf and ear disease has been established. Some varieties show complex resistance, combining reduced susceptibility to Septoria leaf and ear disease. Seed infection with P. nodorum in the regions of Siberia reached 7 thresholds and was largely (52.5 %) determined by the August weather conditions. The study of the collection of spring wheat varieties from three Siberian regions has revealed the following trend. Transmission of P. nodorum with the seeds of varieties was the most active (7.6 %) in Novosibirsk Region and somewhat weaker in Omsk Region (5.7 %). The most favorable phytosanitary situation was in Kurgan Region, where varieties transmitted P. nodorum to a low degree (2.1 %), below the threshold.

## Abstract (ru)

Септориоз листьев и колоса яровой пшеницы – одна из наиболее экономически значимых инфекций в Сибирском регионе. В системах контроля септориоза основным экологически безопасным элементом являются устойчивые сорта, которые тормозят или останавливают развитие эпифитотического процесса путем замедления размножения возбудителей септориоза. Цель работы состояла в уточнении видового состава возбудителей септориоза по регионам Западной Сибири и сортам яровой пшеницы, исследовании эпифитотического процесса септориоза дифференцированно на листьях и колосьях сортов, а также в оценке активности семенной передачи Рarastagonospora nodorum. Исследования проводили в 2016–2018 гг. по общепринятым методикам. В Западной Сибири септориоз листьев и колоса яровой пшеницы широко распространен: 35 % по показателю развития болезни и 90 % по распространенности. Видовой состав возбудителей септориоза представлен Р. nodorum, Septoria tritici и P. avenae f. sp. triticae, причем соотношение видов изменялось по регионам. В Новосибирской области отмечено полное доминирование Р. nodorum, S. tritici встречался в 13.8 раза реже, а P. avenae f. sp. triticae единично. В Тюменской области доминирование Р. nodorum не было таким безусловным и нарушалось в некоторых географических пунктах S. tritici и P. avenae f. sp. triticae. В Алтайском крае во всех точках учета преобладал Р. nodorum; S. tritici и P. avenae f. sp. triticae встречались повсеместно, но в 5.6 и 8.6 раза реже соответственно. Изучение сортов яровой пшеницы разного происхождения не позволило выявить иммунных к септориозу листьев и колоса форм. Установлено дифференцированное проявление признаков устойчивости к септориозу листьев и колоса, выделены сорта, сочетающие пониженную восприимчивость к септориозу и листьев, и колоса. Инфицированность семян фитопатогеном в регионах Сибири достигала 7 экономических порогов вредоносности и в значительной степени (52.5 %) определялась погодными условиями августа. Изучение коллекций сортов из трех регионов Сибири позволило выявить следующую тенденцию: наиболее активно передача P. nodorum с семенами сортов шла в Новосибирской области (7.6 %), в меньшей степени – в Омской (5.7 %); самая благополучная фитосанитарная ситуация была в Курганской области: сорта передавали P. nodorum в слабой степени (2.1 %), ниже экологического порога вредоносности.

## Introduction

Septoria blotch of leaves and ears has long been one of the most common and damaging diseases of spring wheat in all areas of its cultivation (Eyal, 1999; Robert et al., 2004; Nazarova et al., 2010). When wheat is affected by Septoria blotch, the leaves dry out prematurely, and grain is poured only at the expense of the stem and spike green parts. Grain is formed hollow with a low grain unit and 1000-grain mass. The grain productivity of spring wheat falls by 25–60 %. The germination ability and germination energy of seeds are reduced by 7–12 % (Chulkina, 1991; Parker et al., 2004; Robert et al., 2004; Sanin et al., 2015).

are the fungi Parastagonospora nodorum (Berk.) Quaedvl., Verkley & Crous (syn. Depazea nodorum Berk.) and Septoria tritici Roberge ex Desm. (syn. Zymoseptoria tritici (Roberge ex Desm.) Quaedvl. & Crous.). Of the two species of fungi in spring wheat in Siberia, P. nodorum is predominant, characterized by faster (8–10 times) germination of pycniospores and colonization of the host plant tissue compared to S. tritici (Chulkina, 1991). Also, Phaeosphaeria avenaria f. sp. triticae Shoem. & C.E. Babc. (syn. S. avenae f. sp. triticea) is detected in spring wheat in Siberia (Toropova et al., 2019). The same species cause most damage to winter wheat crops (Kolomiets et al., 2018). Each of the plant pathogens has certain epidemiological features and requirements for environmental conditions, which ensures a greater ecological plasticity of the disease and difficulty in its control. Both species can exist together on the same plant. S. tritici mainly affects the leaves, developing more intensively on young than on old tissues. P. nodorum equally well affects both leaves and ears, and is able to live and multiply on dead tissues (Kolomiets et al., 2018). The highest susceptibility to P. nodorum was noted in the phase of heading and flowering (Cooke, Jones, 1970). The phase of the highest sensitivity of wheat to S. tritici occurs during the tillering–bumping period, which is mainly associated with increased air humidity in the crop folia (Adolf et al., 1993). The representation of the main pathogens in the pathogenic complex of winter wheat Septoria blotch is dependent on the weather. S. tritici predominates in years with low winter temperatures and warmer and rainy conditions in the first half of summer. S. nodorum prevails in years with wetter autumn, warm winters, and high rainfall in the second half of summer (Sanin et al., 2017).

Septoria leaf and ear blotch is recorded in more than fifty countries, mainly at latitudes with a temperate climate (Europe, North America, and Australia). In the territory of the former Soviet Union, Septoria is especially prevalent in the North Caucasus, the Urals, the Ukraine, and Belarus (Nazarova et al., 2010).

The germ tubes of plant pathogens are introduced into the leaf tissue of susceptible hosts after the germination of pycniospores in most cases through the stomatal cleft, less often through the epidermis. Several sprouts often penetrate one stoma. After the penetration of pathogens, weak branching and growth of fungi in the intercellular spaces of leaf mesophyll cells is noted, with the majority of hyphae growing along the leaf between the epidermal and mesophyll cells. This, apparently, explains why Septoria blotch spots are often elongated along the leaf veins. Branching of the hyphae gradually occurs, as well as their growth in various directions; the leaf thickness is penetrated several times; and the intercellular spaces are filled with mycelium (Robert et al., 2004; Nazarova et al., 2010). The toxin ochracin, which suppresses the growth of host plants, and septorin, which inhibits oxidative phosphorylation in plant cells, were detected in S. tritici, the Septoria leaf blotch pathogen (Eyal, 1999).

Plant infection with Septoria blotch is particularly successful if the period of drip wetting at the optimum temperature is at least 8 hours and the relative humidity is 98–100 %. Therefore, Septoria blotch most often develops in areas with sufficient moisture. However, there are cases when Septoria blotch is dangerous in dry areas. This is because pathogens can use an intermittent wet period, as a result of regular dewfall (Toropova et al., 2002).

The incubation period of Septoria blotch, depending on hydrothermal conditions, is from 6 to 49 days. The regression analysis showed that 45 % of the variability of the latent period of the pathogen is due to the influence of temperature, 12 % due to its population density, and only 3 % to the duration of hydration (Chulkina, 1991). This indicates that, once in the ecological niche, the pathogen is almost independent of moisture. However, in the external environment throughout all the phases of the transmission mechanism (separation from the causative agent source, transmission of propagules with airborne droplets, and germination and incorporation into the tissues of susceptible plants), its life cycle largely depends on the presence of droplet-liquid moisture (Nolan et al., 1999; Toropova et al., 2002; Nazarova et al., 2010; Pakholkova, 2015).

The reproductive potential of Septoria blotch pathogens is quite high, amounting to 10–15 thousand spores in one pycnium. A close correlation was established between the numbers of pycnia and spores in them (r = 0.901) (Chulkina, 1991).

For the pycnia formation, high relative humidity (over 98 %) is required. The formation of mature P. nodorum pycnia takes 8–14 days, and S. tritici 14–20 days after inoculation (Pakholkova, 2015). At the end of the growing season of host plants, the pycnia number reaches its maximum value. From 6 to 12 fungi generations develop during the season. At the end of the growing season, pycnia and sacs (fruiting bodies) of different species of Septoria blotch pathogens are formed on the same leaves (Kolomiets et al., 2018).

The Septoria blotch pathogens winter on infected plant debris as mycelium, fruiting bodies, and pycnia; P. nodorum also on or inside seeds as mycelium and pycnia (Toropova et al., 2016; Sanin et al., 2018). The pathogens can survive for 6–18 months on infected plant debris in the surface soil layer or on its surface and until the June–July end in the soil at the depth of the arable layer. At the same time, 1 g of plant residues contains 1.5–6 million pathogen spores in the soil and 52–63 million on the soil surface. The viability of both spores in pycnia and especially ascospores in sacs is high, reaching 100 % in the spring wheat earing phase, when mass plant infection occurs (Chulkina, 1991; Toropova et al., 2002; Sanin et al., 2018).

The ability of pycniospores to spread after release from pycnia is associated with rain. Wind without rain cannot take away spores, as they are covered with an adhesive mass, which in the absence of moisture sticks them to the substrate. As you move away from the infection source, the spore population decreases. Further than 500 m away, spores are usually not detected. In the vertical direction, spores in the mass rise to 75 cm and are absent at a height of 150 cm (Eyal, 1999; Toropova et al., 2002; Robert et al., 2004; Sanin et al., 2018).

Ascospores can be released from perithecia only during rain. This process is extended and can last several months. Ascospores are carried over a few (2–3) kilometers.

The Septoria blotch development is largely dependent on hydrothermal conditions. At low humidity, spores are not released from the pycnia and do not spread. Therefore, the disease outbreaks occur in years with significant rainfall, with a maximum air temperature of no higher than 30 °C and an average daily temperature between 14 and 21 °C. The damage by root rot even in a slight degree (3–10 %) enhances the Septoria blotch severity (Toropova et al., 2002; Toropova, 2005; Nazarova et al., 2010; Sanin et al., 2015).

A decrease in soil cultivation intensity and the accumulation of infected plant residues on the soil surface have led to a 2–2.5-fold increase in the frequency of Septoria blotch epiphytotic in the forest-steppe of West Siberia over the past 10 years. Since the seed transmission of the main causative agent P. nodorum has intensified in spring and winter wheat in the last decade, this annually created the prerequisites for the formation of early foci of the disease (Toropova et al., 2018). The epiphytotic development of Septoria blotch foci, in which the infection on the upper leaves reaches the economic threshold (15–20 %), occurs when 3 times more rain falls over the ten-day period than the long-term average, at a temperature of 14–22 °C; the disease develops at a rate of up to 2–3 % per day, which necessitates the use of fungicides (Toropova, 2005; Sanin et al., 2015).

The seed transmission of P. nodorum causes the early appearance of Septoria blotch on the coleoptile and basal leaves in the seedling–tillering phase. There is no linear relationship between the infection of seeds and seedlings. The seed infection by 5–10 % can already lead to an epiphytotic of Septoria blotch in favorable weather conditions (Chulkina, 1991; Sanin et al., 2015; Toropova et al., 2018).

The Septoria blotch pathogens have an r life cycle strategy. The r-strategy characteristic features are: numerous (6–12) generations of conidial sporulation (pycnia with spores) under favorable conditions; high pathogen transmission rate; and a polycyclic, variable type of the epiphytotic process dynamics. The strategy of phytosanitary measures against Septoria blotch is to reduce the rate of pathogen propagation and the development of epiphytotic process to a level below the economic threshold. This is achieved due to the genetic and physiological plant resistance and to the prevention of vertical transmission of P. nodorum with seeds.

The breeding of resistant varieties is the most promising and environmentally friendly component of systems of integrated protection from Septoria leaf and ear blotch of spring wheat. In practice, selection of wheat for resistance to Septoria blotch is difficult, because this trait is unstable, varies in time and space, and is controlled by many mechanisms (Kolomiets et al., 2018). Genotypes with complex resistance are rare: varieties can be resistant to one pathogen type and susceptible to another (Jenkins, Jones, 1981). At one time, it was believed that wheat was generally not resistant to P. nodorum (Scharen, Krupinsky, 1970; Broennimann, 1975). However, further studies showed that the situation is not so obvious (Mullaney et al., 1981; Du et al., 1999).

Stable progress is observed in the selection of wheat varieties resistant to S. tritici. Russian researchers have identified a number of varieties that are recommended for inclusion into the breeding programs as sources and donors of resistance to the pathogen (Kolomiets et al., 2018).

Resistance to Septoria blotch can be either quantitative (horizontal) or isolate-specific (vertical) (Tyryshkin, Ershova, 2004; Kolomiets et al., 2017). Currently, 17 genes of resistance to S. tritici (Stb1–Stb17) have been identified in wheat. Due to genetic analysis in the “wheat–M. graminicola” pathosystem, gene-for-gene interaction has been proven (Kolomiets et al., 2017). Recent studies have established some biochemical mechanisms of resistance of common wheat to Septoria blotch (Veselova et al., 2018, 2019). Also, Septoria-resistant common wheat forms have been isolated abroad (Van Ginkel, Rajaram, 1999; Simón et al., 2003; Robert et al., 2004). One of the aspects that impede the search for plant forms resistant to Septoria blotch is the underestimation of the multicomponent species composition of Septoria blotch causative agents and the insufficient knowledge of the regional species representation in the disease pathogenic complex. In addition, when assessing plant resistance, they often do not carry out a differential account of leaf and spike lesions, though they might be determined by different mechanisms.

The purpose of the work was to clarify the species composition of Septoria blotch pathogens for regions of West Siberia and spring wheat varieties, to study the epiphytotic process of Septoria blotch differentially on the leaves and ears of varieties, and to evaluate the activity of seed transmission of P. nodorum.

## Materials and methods

The studies were carried out in 2016–2018 in the West Siberian forest-steppe zone. Septoria leaf and ear blotch was recorded using the international scale (Chulkina et al., 2017) in the technological conditions of the region’s farms. To clarify the species composition of Septoria blotch pathogens, we collected samples of infected wheat plants and plant residues in the fields at the end of the wheat growing season, taking 10–20 infected leaves at each point. To determine the species of fungi, fragments of the diseased tissue with fruiting bodies (pycnia) were placed on glass slides in a water drop, and after 10–15 minutes they were viewed at low magnification. The shape and size of spores emerging from the pycnia determined the species and its percentage in the total number of the pycnia studied (Pyzhikova et al., 1988). The study of Septoria leaf and ear blotch on the spring wheat varieties and variety specimens was carried out on the natural infection background using a collection from the Institute of Cytology and Genetics of SB RAS. The collection consisted of 10 varieties from 5 Russian regions and 13 foreign samples from 8 countries. The area under each variety (variety specimen) ranged from 3 to 10 m^2^ in triplicate.

According to the degree of damage, the varieties were divided into the following groups: 0–5, highly resistant; 6–20 %, resistant; 21–40 %, poorly susceptible; 41–65 %, susceptible; and 66–100 %, highly susceptible (Sanin et al., 2015).

The seed samples for the analysis were taken from the farms of Novosibirsk, Tomsk, and Tyumen Regions as well as Altai Krai and Krasnoyarsk Krai. Seed analysis for P. nodorum infection was carried out by an original method (Chulkina et al., 2017). Over the years, 258 seed samples of 53 spring wheat varieties have been analyzed totally.

In the northern forest-steppe of the Novosibirsk Region, 2016 was dry (Hydrothermal coefficient = 0.81), whereas 2017 and 2018 were wet (Hydrothermal coefficient = 1.26 and 1.33, respectively), which significantly influenced the intensity of the natural infection background.

## Results and discussion

The Septoria blotch monitoring in agrocenoses of winter and spring wheat in Novosibirsk, Tomsk, Kemerovo, Kurgan, and Tyumen Regions and Altai Krai, conducted in 2016–2018, established a widespread distribution of the disease in spring wheat varieties. The disease incidence was from 5 to 35 %, and its severity reached 90 %. By the start of the earing phase, a critical situation aroused in most agrocenoses, which required prompt measures to protect spring wheat from Septoria blotch of leaves and ears, despite a significant diversity of weather conditions, varieties, and technologies for spring wheat cultivation (Toropova et al., 2019).

The first single foci of Septoria blotch on the lower leaves of spring wheat during transmission of the pathogen from infected plant residues were observed in 2016 and 2017 in the last two ten-day periods of June; in 2018, due to late sowing, in the first two ten-day periods of July. Moreover, P. nodorum appeared earlier (June–early July) than S. tritici (late July–August).

A comparison of weather conditions over the years contrasting in the incidence and severity of Septoria blotch shows that moderate and significant intensity epidemics began when 76.0 to 111.0 mm of precipitation fell at an average air temperature of 16.7 °C. The years favorable to Septoria blotch were distinguished by an increase in precipitation during critical periods for plant infection by an average of 6.7 times and a decrease in air temperature by an average of 2.5 °C.

The climatic trend characterized by warming and increased contrast in the weather conditions during the growing season turned out to be favorable to plant pathogens, leading to an increase in the frequency of Septoria blotch epidemics in spring wheat distribution regions, including West Siberia (Levitin, 2015; Toropova et al., 2016). The results of our studies are consistent with published data on the increase in the spread of Septoria blotch on the winter wheat in the European part of Russia (Sanin et al., 2017; Gultyaeva et al., 2019).

Septoria leaf and ear blotch of spring wheat was encountered as P. nodorum, S. tritici, and P. avenae f. sp. triticae, and the ratio of species varied across the regions (Table 1). The table shows that P. nodorum, S. tritici, and P. avenae f. sp. triticae pycnia were present on the infected leaves of spring wheat varieties cultivated in Siberia; however, their ratio in the regions varied significantly. Thus, according to the averaged data, at 6 sampling points in Novosibirsk Region, an overwhelming dominance of P. nodorum was revealed. S. tritici was 13.8 times less common; P. avenae f. sp. triticae in the Septoria blotch pathogenic complex in Novosibirsk Region was a singleton.

**Table 1.**
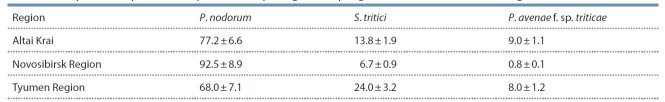
The species composition of Septoria blotch pathogens on spring wheat leaves in the Siberian regions, 2016–2018, %

A study of the species composition of Septoria blotch pathogens in Tyumen Region showed a significant diversity at the sampling points. Two sampling points in Tyumen Region were under the dominance of P. nodorum; the second position belonged to P. avenae f. sp. triticae, not to S. tritici. On the wheat leaves from the third point, only P. nodorum was detected. The dominance of S. tritici was revealed on wheat leaves from the fourth point in Tyumen Region, and at 3 points out of five P. avenae f. sp. triticae made a significant contribution to the pathogenic complex of Septoria blotch in spring wheat, which was not observed in Novosibirsk Region.

P. nodorum dominated at all the sampling points in Altai Krai. S. tritici and P. avenae f. sp. triticae were found everywhere, but 5.6 and 8.6 times less often than the main causative agent of the disease, respectively. P. avenae f. sp. triticae was found on spring wheat leaves in Altai agrocenoses 11.3 times more often than in Novosibirsk Region; that is, its contribution to the Septoria blotch pathogenic complex was much more significant.

Thus, significant differences were found in the species composition of Septoria blotch of spring wheat in regions of West Siberia, and this should be taken into account when creating pathogen populations for artificial infection of plants during selection for resistant varieties. A comparison of the data presented above with the results of similar studies in the 1980s (Chulkina, 1991) indicates that the species composition of Septoria blotch has undergone some changes and has become more diverse in the regions. Note the appearance of P. avenae f. sp. triticae in the pathogenic complex of Septoria blotch at all the sampling points, which was not mentioned 40 years ago. The change in the species composition is probably associated with both climatic variations and a change in the spring wheat cultivating technology.

Table 2 shows the differences in the species composition of Septoria blotch on the spring wheat varieties from the Institute of Cytology and Genetics collection in Novosibirsky District of Novosibirsk Region. The table shows that three Septoria blotch pathogens were present on the spring wheat leaves: P. nodorum, S. tritici, and P. avenae f. sp. triticae. The main causative agent of Septoria leaf and ear blotch was P. nodorum, the occurrence of which averaged 85.4 % of the pathogenic complex in all the varieties. The second place in distribution on the wheat leaves was taken by S. tritici pycnia, 11.8 %, which reached a maximum of 20 % in the varieties ‘Kaiyr’ and ‘KWS Akvilon’. The pathogen of the most limited occurrence was P. avenae f. sp. triticae: it was detected in only 8 varieties, and the average occurrence was 2.8 %. The data obtained indicate a predominantly regional confinement of the pathogen species composition. The varieties of different origins were infected with plant pathogens according to the regional type that is characteristic of Novosibirsk Region.

**Table 2.**
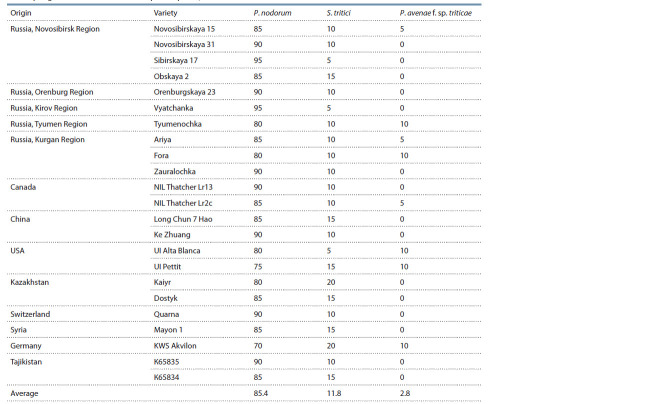
The species composition of causative agents of Septoria blotch in the spring wheat varieties in the full ripeness phase, %

Assessment of the resistance of 23 spring wheat varieties to Septoria leaf and ear blotch in the northern forest-steppe of the Ob (Novosibirsk) region showed the absence of plant forms immune to Septoria blotch (Table 3).

**Table 3.**
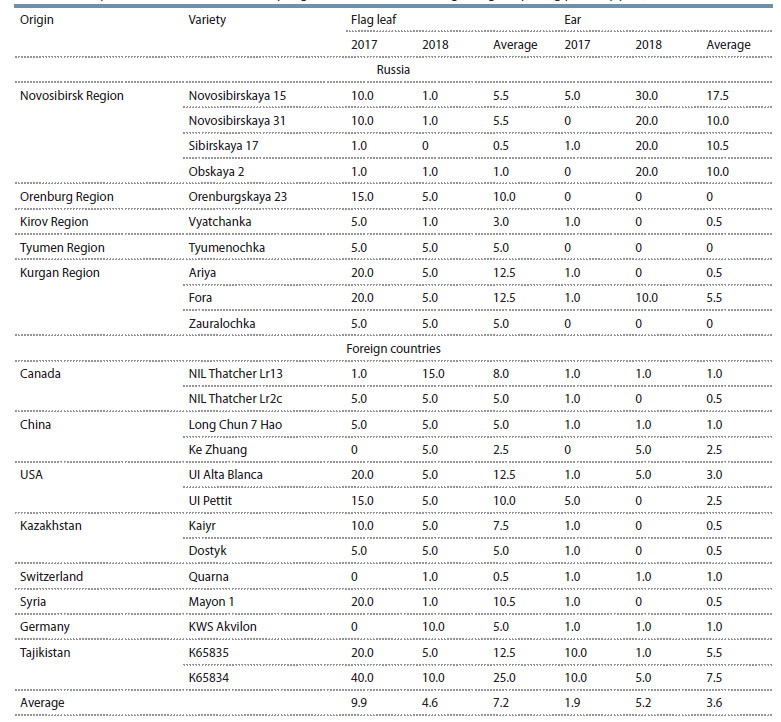
The Septoria blotch incidence in the spring wheat varieties at the beginning of ripening phase by year, %

The formation of Septoria blotch foci each year began on cereal grasses, such as common meadow-grass and cock’sfoot, in which the disease severity by the first detecting date (July 5–7) had already reached 60 %. By that time, on winter wheat, the Septoria blotch symptoms had been detected on the second and third leaves from above and averaged 10 %. On the spring wheat, the Septoria blotch signs were noted only on the lower leaves and reached 3 % in the first ten-day period of July.

The years of research were wet, and the weather was favorable to the disease development. At the beginning of the loading phase, all the studied varieties showed signs of Septoria blotch infection; however, the intensity of the epiphytotic process varied significantly, both by variety and by plant organs in both years of research. For example, in 2017, the disease incidence ranged from 0 to 40 % on the flag leaves of spring wheat varieties and from 0 to 10 % on the spikes. In 2018, flag leaves were affected in the same varieties more

evenly, from 0 to 15 %, and the wheat ears, in comparison, showed stark contrast, from 0 to 30 %. The tendency of differentiated manifestation of resistance to Septoria blotch on the leaves and ears of varieties was revealed. The correlation coefficient of Septoria blotch of leaves and spikes by variety was r = 0.414 ± 0.280.

‘Sibirskaya 17’ and ‘Obskaya 2’ (Novosibirsk) were the most resistant to Septoria leaf blotch, with moderate spike damage. In both years of research, the flag leaf was affected at a sporadic level, providing grain loading. However, the resulting grain could become infected with P. nodorum and lead to the appearance of early Septoria blotch foci when sowing seeds in the following year.

Resistance to Septoria spike blotch was shown by ‘Orenburgskaya 23’ (Orenburg Region) and ‘Ariya’ (Kurgan Region), as well as the foreign varieties ‘NIL Thatcher Lr13’ (Canada), ‘Kaiyr’ (Kazakhstan), ‘Mayon 1’ (Syria), and ‘KWS Akvilon’ (Germany), with either unaffected or sporadically affected ears during the loading phase in both years of research. Flag leaves in these varieties were affected by Septoria blotch at the level of 10–20 %.

The domestic varieties ‘Vyatchanka’ (Kirov Region), ‘Tyumenochka’ (Tyumen Region), and ‘Zauralochka’ (Kurgan Region) and the foreign varieties ‘NIL Thatcher Lr2c’ (Canada), ‘Long Chun 7 Hao’ and ‘Ke Zhuang’ (China), ‘Dostyk’ (Kazakhstan), and ‘Quarna’ (Switzerland) showed complex resistance to both Septoria diseases, leaf blotch and ear blotch. This group of varieties was slightly affected by Septoria leaf and ear blotch, and the domestic varieties ‘Tyumenochka’ and ‘Zauralochka’ had a completely healthy spike at the beginning of the filling phase with a weak damage to the flag leaves.

The survey carried out in the phase of milk ripeness showed that the Septoria blotch severity reached 100 % in all varieties. The domestic varieties ‘Orenburgskaya 23’ and ‘Vyatchanka’ as well as ‘Long Chun 7 Hao’ from China showed a complex decreased susceptibility in the phase of milk ripeness. They had moderate, at the level of economic threshold (20 %), leaf and spike damage at the end of the growing season.

The variance analysis showed that the influence of the year conditions on the incidence of Septoria leaf blotch was 17.9–25.4 % (1 % significance level). The influence of the variety factor was 3.5–10 times lower and not always statistically significant.

Considering the ability of P. nodorum to use seeds as a transmission factor in time and to create early disease foci, we evaluated the intensity of seed infection in spring wheat varieties. The results of monitoring seed infection with P. nodorum in the Siberian regions are presented in Table 4.

**Table 4.**
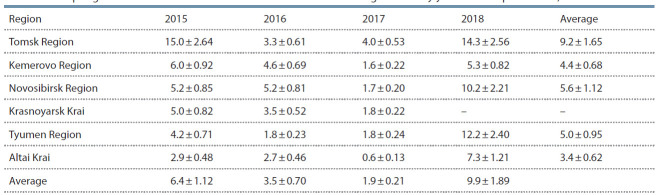
The spring wheat seeds infection with P. nodorum across Siberia regions and by years of seed production, % Note: Influence of the factors: “region” – 15.1 % (level of significance 5 %); “year” – 52.5 % (level of significance 1 %).

The maximum infection of seed samples with Septoria blotch in the spring of 2016 (the year of seed production 2015) was noted in Tomsk Region, where it reached 36 %, which is more than 7 economic thresholds (Chulkina et al., 2017). In Krasnoyarsk Krai, seed infection reached 2.5 thresholds; in the remaining regions it was about 2 thresholds. The exception was Altai Krai, where in 2015 there were favorable conditions for obtaining high-quality seeds and the maximum infection rate reached 7 %.

The analysis of spring wheat seeds in the spring of 2017 showed that the infection of individual samples with Septoria blotch reached 4.4 thresholds, while the average seed infection only in Novosibirsk Region reached the threshold level. The remaining regions provided moderately infected seeds for analysis, on average below the economic threshold.

According to spring studies in 2018, the infection of spring wheat seeds with P. nodorum reached the economic threshold only in individual samples, while the average in all regions studied did not reach the economic threshold. The highest seed infection was noted in the more humid Tomsk Region. The most favorable situation for Septoria blotch was revealed in Altai Krai, where grain ripening in summer and September, 2017, in most areas took place under the dry weather. In general, the infection of the spring wheat seeds for sowing in 2018 was insignificant, lower than that in the previous years.

P. nodorum was detected in significant quantities on the spring wheat seeds produced in 2018 in all the regions. The average seed infection exceeded the economic threshold. On the seeds from Tomsk and Tyumen Regions the P. nodorum infection reached 7 thresholds, which should be considered a strong epidemic (Chulkina, 1991; Toropova et al., 2002). In 70 % of the seed samples the Septoria blotch threshold was exceeded, and by 38.5 % it was exceeded more than 2 times. In most West Siberian regions the 2018 growing season was quite moist, characterized by the epidemic severity of Septoria leaf and ear blotch, which provided P. nodorum with favorable conditions for seeds infection. In general, the 2019 spring analysis revealed the highest seed infection in recent years, which created the prerequisites for the early occurrence of a Septoria blotch epiphytotic process in all the regions of Siberia. The variance analysis showed that the influence of the factor “region” on the infection of spring wheat seeds with P. nodorum, reflecting the climate and cultivation technologies, is 3.4 times weaker than the influence of the factor “year weather conditions”. In the more humid Tomsk Region the infection of spring wheat seeds with the water-depending pathogen P. nodorum has been 2 times higher on average over the years, compared to the least moistened Altai Krai, where the spring wheat grain production is concentrated mainly in arid warm zones. The correlation coefficients between P. nodorum seed infection and the total precipitation in August were r = 0.746 ± 0.135 to 0.872 ± 0.126 by year and region (5 % significance level). The data presented indicate that it has relevance to the control of vertical transmission of P. nodorum with seeds of spring wheat varieties.

The analysis of spring wheat seeds from the breeding plots in Novosibirsk, Kurgan, and Omsk Regions (Table 5) indicates some differences in the activity of seed transmission of P. nodorum in years favorable for Septoria blotch. All the varieties from the Institute of Cytology and Genetics collection (Novosibirsk Region) ensured the transmission of pathogenic fungus at the level of economic threshold or 2.4 times higher; no varieties resistant to vertical transmission were detected. The varietal difference in the activity of vertical transmission of P. nodorum reached 2.4 times. In Kurgan Region the varieties transmitted P. nodorum 3.8 times more weakly; in none of the varieties did the seed infection reach the threshold. The varietal differences in seed infection reached 3 times. In Omsk Region the situation was intermediate: the transmission of the plant pathogen with seeds was on average 25 % less active than that in Novosibirsk Region and 2.7 times more active than that in Kurgan Region. In the collection of the Omsk State Agrarian University 4 varieties were identified in which the transmission of Septoria blotch was at or up to 1.8 times above the threshold. The varietal differences in the studied parameter reached 2.3 times.

**Table 5.**
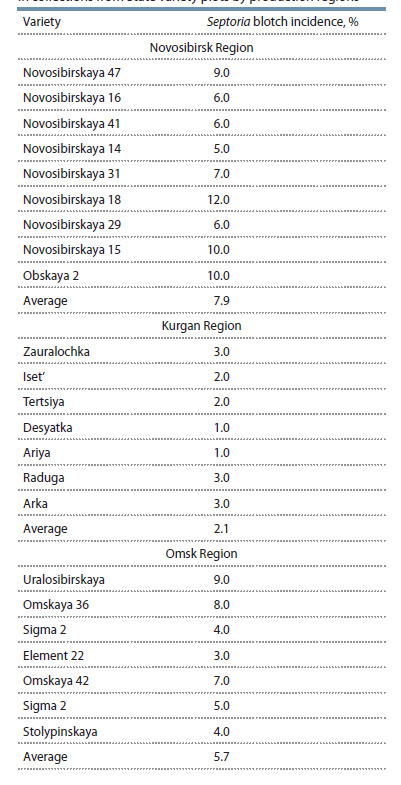
The spring wheat seed infection with P. nodorum in collections from State variety plots by production regions

## Conclusion

Many years of studies have shown that Septoria leaf and ear blotch of spring wheat is widespread in the Siberian regions, reaching 35 % in terms of the disease incidence and 90 % in severity, which indicates the relevance of breeding resistant varieties. When developing breeding programs the species composition of Septoria blotch pathogens (P. nodorum, S. tritici, and P. avenae f. sp. triticae) should be taken into account – all the more so because it is characterized by significant regional differences. In Novosibirsk Region, P. nodorum completely dominated; S. tritici was 13.8 times less common; and P. avenae f. sp. triticae was a singleton. In Tyumen Region, the dominance of P. nodorum was disrupted in some geographic locations by S. tritici and P. avenae f. sp. triticae. In Altai Krai, P. nodorum predominated at all points studied; S. tritici and P. avenae f. sp. triticae were found everywhere, but 5.6 and 8.6 times less often, respectively.

Modern spring wheat varieties of different origins do not have complete immunity to Septoria blotch, but are characterized only by resistance or poor susceptibility to the disease. An independent manifestation of resistance to Septoria leaf blotch and Septoria ear blotch has been established. The correlation coefficients for the incidence of Septoria leaf and ear blotch have been r = 0.323 ± 0.241 to 0.414 ± 0.280 over the years and varieties. Some varieties show relative resistance to Septoria leaf blotch with severe damage to the ear; others, in contrast, are resistant to Septoria ear blotch with severe damage to the leaf apparatus. Based on these data a cautious assumption can be made about the different genetics of resistance to Septoria leaf and ear blotch. The varieties ‘Orenburgskaya 23’ (Orenburg Region) and ‘Ariya’ (Kurgan Region), as well as the foreign varieties ‘NIL Thatcher Lr13’ (Canada), ‘Kaiyr’ (Kazakhstan), ‘Mayon 1’ (Syria), and ‘KWS Akvilon’ (Germany), show complex decreased susceptibility to the disease. They are weakly affected by Septoria leaf and ear blotch. The domestic varieties ‘Tyumenochka’ (Tyumen Region) and ‘Zauralochka’ (Kurgan Region) have a completely healthy ear at the beginning of the filling phase with weak damage to the flag leaf and can be considered the most promising sources of resistance.

The activity of vertical transmission of P. nodorum with seeds should also be monitored in the selection process, since seed transmission of Septoria blotch increases the infectious load on the plants significantly. Seed infection with the pathogen in the Siberian regions reached 7 thresholds and was largely determined by the weather conditions in August. The study of the collection of spring wheat varieties from three Siberian regions has revealed the following trend: transmission of P. nodorum with the seeds of varieties was the most active (7.6 %) in Novosibirsk Region and somewhat weaker in Omsk Region (5.7 %). The most favorable phytosanitary situation was in Kurgan Region, where varieties transmitted P. nodorum in a weak degree (2.1 %), below the threshold.

## Conflict of interest

The authors declare no conflict of interest.
